# Timing of radiotherapy and autologous stem cell transplantation in embryonal tumor with multilayered rosettes: a critical narrative review

**DOI:** 10.1007/s00381-026-07490-8

**Published:** 2026-10-09

**Authors:** Sudarshawn Damodharan

**Affiliations:** Division of Pediatric Hematology and Oncology, University of Chicago, Chicago, IL USA

**Keywords:** ETMR, Autologous stem cell transplantation, Radiotherapy timing, High-dose chemotherapy, Pediatric brain tumor, Pseudoprogression

## Abstract

**Purpose:**

Embryonal tumor with multilayered rosettes (ETMR) carries a median overall survival of approximately 10–12 months despite maximal-safe resection, high-dose chemotherapy (HDCT) with autologous stem cell transplantation (ASCT), and radiotherapy (RT). RT has historically been deferred or omitted in these predominantly infant-age patients owing to concerns over neurodevelopmental toxicity, with HDCT/ASCT serving as the principal consolidation strategy. More recent data have called this approach into question. This narrative review synthesizes the evidence on the timing of RT relative to HDCT/ASCT and its association with outcome in ETMR.

**Methods:**

A structured narrative review of PubMed, EMBASE, and the Cochrane Library (2000–2025) identified studies reporting ETMR-specific outcomes with extractable data on RT timing relative to ASCT. Major prospective trials (ACNS0334 and P-HIT/HIT2000) and institutional or registry series (*n* ≥ 4 molecularly or histopathologically confirmed ETMR patients) were considered.

**Results:**

Nine studies or study-level cohorts providing ETMR-specific RT-timing data were identified, including the Rare Brain Tumor Registry cohort of 159 molecularly confirmed patients. Across independent cohorts, early RT administered within approximately six weeks of surgery or before protracted HDCT/ASCT is consistently overrepresented among long-term survivors (> 36 months): 10 of 26 (38%) in one literature survey and near-universal RT (17/18; 94%) in another. In the Rare Brain Tumor Registry, two-year event-free survival after gross total resection was 66% with HDCT plus RT versus 21% with HDCT and no RT, and 0% with conventional chemotherapy without RT. Radiation-sparing protocols (ACNS0334) showed predominantly local failure (88%), with salvage RT after progression providing no durable benefit. Tandem HDCT/ASCT without upfront RT (P-HIT/HIT2000) produced a five-year OS of 47% for the carboplatin–etoposide plus tandem-HDCT backbone, superior to other chemotherapy regimens but with frequent on-treatment relapse and no independent prognostic benefit demonstrable for radiotherapy as delivered. Combined RT and HDCT/ASCT was associated with improved survival in some series but, in a small series treated with tandem ASCT, with a high incidence of pseudoprogression and neurocognitive impairment.

**Conclusions:**

Current evidence indicates that RT deferral beyond the HDCT/ASCT consolidation window is associated with inferior local control and worse long-term outcomes in ETMR. PNOC031, a prospective but nonrandomized trial that assigns RT-eligible patients to early focal RT and RT-ineligible patients to HDCT/ASCT in parallel cohorts, together with future prospective studies, should help to resolve this. Pending those results, and recognizing that the supporting data are observational and subject to selection bias, early focal RT following gross-total resection appears a reasonable and defensible strategy in eligible patients. The relationship between RT timing, HDCT/ASCT, pseudoprogression, and neurodevelopmental toxicity is not yet well understood and warrants further study.

## Introduction

Embryonal tumor with multilayered rosettes (ETMR) is a distinct and uniformly aggressive central nervous system (CNS) malignancy of early childhood. First characterized as a molecular entity through amplification at the chromosome 19q13.42 microRNA cluster (C19MC) and hallmark expression of LIN28A [2, 4], ETMR achieved independent molecularly defined status in the 2021 World Health Organization Classification of Tumors of the Central Nervous System [1]. The classification consolidates three previously separate histological diagnoses of embryonal tumor with abundant neuropil and true rosettes (ETANTR), medulloepithelioma, and ependymoblastoma under a unified molecular umbrella, acknowledging their shared biology and equivalent clinical behavior [3, 5].

ETMR is characterized by extreme rarity and early onset. The age-adjusted incidence has been estimated at 1.35 per million children aged 1–4 years, with a median age at diagnosis of 24–37 months across published series; in the largest molecularly confirmed registry cohort, the median age was 26 months [7, 9]. The tumor predominates in the supratentorial compartment, though infratentorial and pineal presentations occur, and although leptomeningeal dissemination is recognized, extracranial spread is rare [31]. Despite maximal-safe resection, intensive multimodal chemotherapy, and RT, median overall survival (OS) approximates 10–12 months, and five-year OS across published cohorts rarely exceeds 30% [5, 7–9].

The central therapeutic dilemma in ETMR is well established as curative intent requires RT, yet the patients who require it most are the youngest and most vulnerable to radiation-induced neurocognitive injury. High-dose chemotherapy (HDCT) with autologous stem cell transplantation (ASCT) has therefore been incorporated into most protocols with a dual purpose to intensify systemic treatment and, critically, to serve as a temporizing strategy permitting delay or avoidance of radiation. This approach, drawn from successful models in infant medulloblastoma [10–12], has shaped the design of multiple prospective trials over the past two decades.

The timing of RT relative to HDCT/ASCT has, however, never been subject to a dedicated prospective analysis. Existing data derive from retrospective institutional series, literature surveys, registry analyses, and subgroup analyses of heterogeneous trials, contexts in which ETMR patients are often outnumbered by medulloblastoma and other embryonal histologies. The recently opened PNOC031 international trial, which stratifies patients by RT eligibility and prospectively assigns early focal RT to eligible patients, is the first trial designed to address this question directly.

**Fig. 1:**
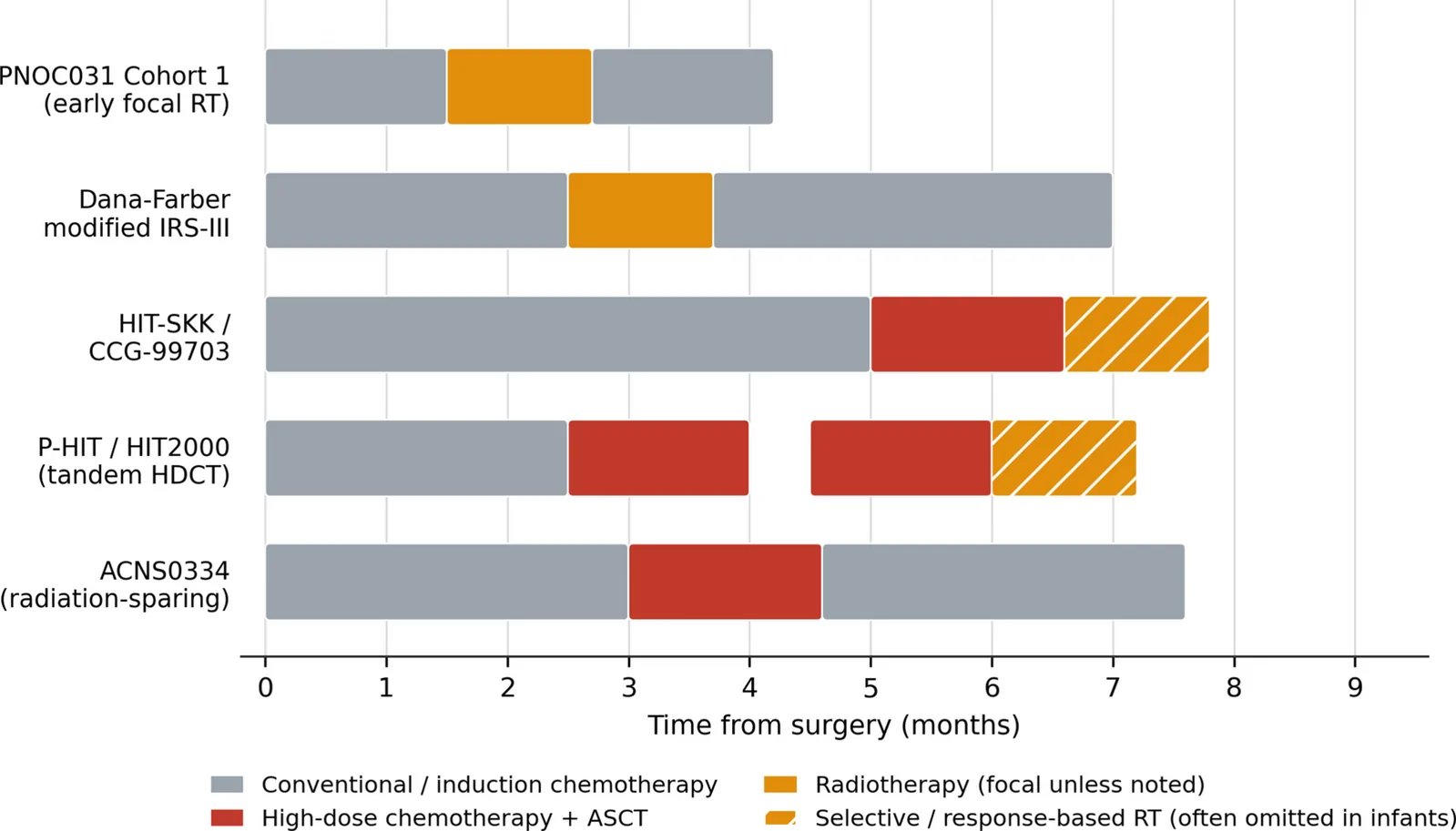
Schematic of radiotherapy timing relative to HDCT/ASCT across major ETMR protocols. Horizontal bars represent time from surgery (months). Gray = conventional/induction chemotherapy; red = high-dose chemotherapy with ASCT; orange = focal radiotherapy; hatched orange = selective or response-based radiotherapy, frequently omitted in infants. PNOC031 cohort 1 delivers early focal RT after a short induction, in contrast to the protracted pre-RT interval imposed by tandem HDCT/ASCT in HIT-SKK/CCG-99703 and P-HIT/HIT2000, or the absence of mandated RT in ACNS0334

This narrative review summarizes the evidence on RT timing in relation to HDCT/ASCT in ETMR and the patterns that recur across the available studies. We consider the mechanisms by which RT deferral may compromise outcome, describe the pseudoprogression that appears specific to the combination of RT and HDCT/ASCT in this disease, and outline the questions that PNOC031 and subsequent trials will need to address (Fig. 1).

## Methods

### Search strategy

A structured search was conducted across PubMed, EMBASE, and the Cochrane Library in March 2025. Search terms combined controlled vocabulary and free-text terms for the entity (“ETMR”, “embryonal tumour with multilayered rosettes,” “ETANTR,” “medulloepithelioma,” “ependymoblastoma,” “C19MC,” and “LIN28A”) with terms for treatment modalities (“radiotherapy,” “radiation therapy,” “high-dose chemotherapy,” “autologous stem cell,” “ASCT,” “HDCT,” and “stem cell transplant”). No language restriction was applied. Publication dates from January 2000 to March 2025 were included to capture the period following initial molecular characterization of the entity. Reference lists of identified publications were hand-searched for additional studies.

### Study selection

Studies were included if they (1) enrolled patients with histopathologically and/or molecularly confirmed ETMR (including pre-2021 diagnoses retrospectively reclassified by C19MC/LIN28A status); (2) reported survival outcomes including OS and/or event-free survival (EFS) or progression-free survival (PFS); and (3) provided sufficient information to characterize RT timing in relation to HDCT/ASCT delivery. Studies including ETMR as a subgroup within broader embryonal tumor cohorts were included where ETMR-specific outcome data could be extracted. Single-patient case reports were excluded unless forming part of a systematic literature survey.

### Data extraction

For each study, the following were extracted: study design, number of ETMR patients, chemotherapy protocol, HDCT/ASCT approach (single versus tandem and conditioning regimen), RT approach (craniospinal versus focal andphoton versus proton), timing of RT relative to HDCT/ASCT, and survival outcomes at defined time points. Definitions of “early” and “deferred” RT varied across studies and are noted where applicable.

## Results

### Overview of identified studies

Nine studies or study-level cohorts providing ETMR-specific RT-timing data were identified (Table 1; protocol-level approaches are summarized in Table 2 and the long-term survivor synthesis in Table 3). These draw from prospective trials (ACNS0334 and P-HIT/HIT2000), the international Rare Brain Tumor Registry [9], retrospective institutional series, literature surveys [17, 18], a multi-institutional proton-therapy registry (PPCR 2024), and a modified IRS-III case series [28]. No randomized controlled trial directly comparing RT-timing strategies has been completed. The quality of evidence is therefore uniformly observational, and all conclusions are hypothesis-generating rather than confirmatory.

**Table 1:** Summary of key published studies reporting radiotherapy timing and ASCT data in ETMR

Study (year)	*N* (ETMR)	Protocol/regimen	RT timing relative to HDCT/ASCT	5-yr OS	5-yr PFS/EFS	Key notes
Juhnke et al. (P-HIT/HIT2000, 2022)	35	CARBO-ETO induction + tandem HDCT; HIT-SKK; RT per response/stage	Deferred; given for persistent disease, relapse, or progression only; no mandated early RT	47% (CARBO-ETO + HDCT) vs 8% (other) in 30-pt analysis cohort; RT not an independent prognostic factor (time-dependent Cox)	33.3% (CARBO-ETO + HDCT)	Best survival with tandem HDCT; RT given selectively
Mayr et al. (2020)	9 (inst.) + 237 (lit.)	Multimodal; HDCT ± ASCT; varied backbones	Early RT (~ 6 wk post-diagnosis, post-GTR) vs deferred (end of chemo)	2/9 survivors at 50–56 mo; both early RT. 11% of 237 lit. pts > 36 mo	NR	All deferred-RT pts recurred. 24/26 LT survivors received RT; 10/26 early after GTR
Jaramillo et al. (2018)	7 (inst.) + 204 (lit.)	Proton RT; varied HDCT/ASCT in subset; lit. incl. photon	Median age 42 mo at RT start; 5/7 progressed before RT	Median OS 16 mo (inst.); 18 lit. LT survivors, 17/18 (94%) received RT	3/7 survived ≥ 36 mo	All 5 who progressed before RT failed in tumor bed; 14/18 lit. LT survivors received CSI
Khan et al. (Rare Brain Tumor Registry, 2021)	159	Multimodal; surgery + multiagent chemo ± HDCT ± RT	Stratified by modality; RT often deferred or omitted in infants	2-yr OS 29% overall; 66% (5-yr) with HDCT + RT after GTR	2-yr EFS 31% overall; 66% (HDCT + RT, GTR) vs. 21% (HDCT, GTR, no RT) vs. 0% (chemo, no RT)	Largest molecularly confirmed cohort; RT-inclusive therapy strongly favoured
ACNS0334 – COG (Ihle et al., 2020) [abstr]	14	Induction ± MTX + HDCT/SCR; no mandated radiation	Radiation withheld pre-progression (radiation-sparing)	4/14 alive w/o progression at 5–10 + yr; 5/14 progressed on Rx	Not calculable; 5/14 progressed on-treatment	88% of progressions local. Salvage RT after progression: all 4 died within 3–13 mo
Gevorgian et al. (2022)	9 (in 50-pt cohort)	Tandem autoHSCT (HIT-MED, 84%); single ASCT (16%); adjuvant RT 50%	RT adjuvant in 25/50; post-ASCT; RT at relapse in 6/50	37% (ETMR); 71% MB; 51% other	Cohort-wide 5-yr PFS 46%	ETMR OS markedly inferior to MB; RT-free OS for MB only 24%
PPCR Registry (Krisch et al., 2025)	20 enrolled (18 eval.)	Focal proton RT (median 54 Gy) in 16/18; CSI in 2 (30.6–36 Gy); 61% HDCT; 9 sites, 2013–2021	Median age at RT 3.0 y; median FU 55.5 mo; timing not significant (≥ 200 d *p* = 0.35)	4-yr OS 59.6%	4-yr EFS 54.2%; 4-yr local control 81%	No significant OS/EFS difference by HDCT, progression before RT, or surgery-to-RT ≥ 200 d; effective local control with focal proton RT; registry captures only RT recipients
Gutierrez-Quintana et al. (2024)	4	CCG 99703 (conventional chemo + HDCT/ASCT); RT off-protocol	RT before HDCT (*n* = 3) or after HDCT (*n* = 1)	3/4 alive at median 50.5 mo (37–64)	All 4 survived > 36 mo	Pseudoprogression and severe neurocognitive impairment in all four survivors (very small series)
Hanson et al. (2020)	5	DanaFarber modified IRS-III (doxorubicin + actinomycin D)	Focal RT in 4/5; 1/5 received HDCT/ASCT instead of RT	4/5 alive, PFS > 18 mo	4/5 PFS > 18 mo	Single HDCT/ASCT patient (no RT) also PFS > 18 mo, but follow-up limited

*ASCT* autologous stem cell transplantation, *CARBO-ETO* carboplatin–etoposide, *COG* Children’s Oncology Group, *CSI* craniospinal irradiation, *EFS* event-free survival, *ETMR* embryonal tumor with multilayered rosettes, *GTR* gross total resection, *HDCT* high-dose chemotherapy, *HIT* Hirntumor trial, *LT* long-term, *MB* medulloblastoma, *MTX* methotrexate, *NR* not reported, *OS* overall survival, *PFS* progression-free survival, *PPCR* Pediatric Proton/Photon Consortium Registry, *RT* radiotherapy, *SCR* stem cell rescue

**Table 2:** Protocol overview: approach to RT timing in major ETMR treatment strategies

Protocol/trial	Era	HDCT/ASCT approach	RT strategy	Age constraints
HIT-SKK/CCG-99703	1990s–2000s	Single or tandem ASCT; thiotepa/carboplatin or busulfan-based conditioning	Deferred until end of HDCT; often omitted in infants	Primarily < 3 yr; radiation avoided if possible
P-HIT/HIT2000	2001–2014	Carboplatin–etoposide induction + tandem HDCT (CARBO-ETO)	Response-/stage-stratified; given for persistent disease, not upfront	Age-stratified; < 18 mo typically no RT
ACNS0334 (COG)	~ 2007–2015	Induction ± high-dose methotrexate + HDCT/SCR	No mandated RT; radiation-sparing intent	Infants (< 3 yr); radiation-avoidance central design principle
HIT-MED/KiKME	2014–ongoing	Tandem ASCT preferred; HIT-MED protocol	Post-ASCT consolidative RT or at relapse; not upfront	Primarily < 4 yr; RT deferred to reduce neurotoxicity
IRS-III modified (DanaFarber)	2015–present	HDCT/ASCT as option if RT not feasible; conventional backbone preferred	Focal RT in majority; HDCT/ASCT as RT substitute in RT-ineligible patients	All ages; RT preferred when feasible
PNOC031 (active)	2024–ongoing	Cohorts 2 and 3: HDCT with ASCT for RT-ineligible patients	Cohorts 1 and 3A: early focal RT after induction; cohorts 2 and 3B: HDCT/ASCT without upfront RT	RT eligibility stratifies cohort; RT age criteria > 12 mo (infratentorial) or > 15 mo (supratentorial)

*COG* Children’s Oncology Group, *HDCT* high-dose chemotherapy, *HIT-MED* HIT medulloblastoma protocol, *HIT-SKK* infant brain tumor protocol (Säuglings- und Kleinkinderkopftumoren), *PNOC* Pacific Pediatric Neuro-Oncology Consortium, *RT* radiotherapy, *SCR* stem cell rescue

**Table 3:** Long-term survivors (> 36 months) by radiotherapy receipt and timing: cross-study synthesis

Source	Total ETMR pts	LT survivors (> 36 mo)	RT received	RT timing	HDCT/ASCT in LT survivors	Inference
Mayr et al. 2020	237 (lit.)	26 (11%)	24/26 (92%)	10/26 early (post-GTR); remainder deferred	Not systematically reported	Early RT post-GTR overrepresented in survivors
Jaramillo et al. 2018	204 (lit.)	18 (9%)	17/18 (94%)	Not specified; mostly with initial treatment	Varied; often combined	Near-universal RT in survivors; 14/18 received CSI
Khan et al. 2021	159	2-yr EFS 66% (HDCT + RT, GTR) vs 21% (HDCT, GTR, no RT)	Curative-intent survival 0% without RT (conventional chemo)	Often deferred/omitted in infants	Subset HDCT + GTR without RT (2-yr OS 30%)	Largest cohort; RT-inclusive therapy strongly favored
ACNS0334 (COG)	14	4 (29%)	0/4 (0%) at initial therapy	N/A—no upfront RT	4/4 received HDCT/SCR	Small subset achieves durable remission with HDCT alone; selection bias cannot be excluded
Gutierrez-Quintana et al. 2024	4	3 (75%) at median 50.5 mo	4/4 (100%)	Pre-HDCT (*n* = 3) or post-HDCT (*n* = 1)	4/4 (tandem ASCT, CCG 99703)	All received both RT and HDCT; RT-timing effect cannot be isolated
PPCR (Krisch 2025)	18 (eval.)	4-yr OS 59.6%; EFS 54.2%; local control 81%	18/18 (registry of RT recipients only)	Median age at RT 3.0 y; no significant difference by surgery-to-RT ≥ 200 d	11/18 received HDCT	No significant survival difference by RT timing or HDCT; focal proton RT enables good local control; RT-only registry limits inference

Long-term survival defined as ≥ 36 months without evidence of disease at last follow-up, consistent with Mayr et al. (2020) and Jaramillo et al. (2018)

*CSI* craniospinal irradiation, *EFS* event-free survival, *GTR* gross total resection, *HDCT* high-dose chemotherapy, *LT* long-term, *OS* overall survival, *RT* radiotherapy

### The radiation-sparing strategy and its failure mode

The biological rationale for deferring RT in ETMR as in infant medulloblastoma rests on the significant, dose-dependent neurocognitive harm attributable to cranial radiation in children under three years of age, particularly when delivered to large brain volumes. Craniospinal irradiation (CSI) in infants has been associated with progressive cognitive decline, neurosensory deficits, and endocrine sequelae that persist and compound over time [13, 14]. Against this background, HDCT/ASCT emerged as a consolidation strategy that could, in principle, eradicate residual disease sufficiently to permit RT deferral or omission—an approach validated in a subset of infant medulloblastoma patients on the Baby POG and CCG-9921 protocols [10, 11].

In ETMR, this radiation-sparing model has failed to replicate the outcomes seen in favorable medulloblastoma subgroups. The clearest evidence comes from ACNS0334, the Children’s Oncology Group (COG) phase III trial that enrolled children younger than 36 months with high-risk embryonal brain tumors and delivered three cycles of induction (randomized to include high-dose methotrexate or not) followed by three cycles of high-dose consolidation chemotherapy with stem cell rescue, with no radiation mandated [16]. Retrospective molecular analysis identified 14 patients within the ACNS0334 cohort as ETMR (20% of the molecularly analyzed cohort), predominantly female, largely nonmetastatic (10 M0, 1 M1, and 3 M2/M3) and arising in the cerebrum (8), cerebellum (3), and pineal gland (3); gross total resection was achieved in 5 of 11 patients with M0/M1 disease. Methotrexate conferred no benefit in this subgroup (event-free survival 20.0% with versus 33.3% without). Of these, five progressed on treatment, with 88% of progressions occurring at the primary tumor site, consistent with inadequate local control. Among those irradiated for salvage following progression, all four patients died within 3 to 13 months. Although four ETMR patients achieved durable remission, surviving 5 to more than 10 years without radiation, this minority may reflect a biologically distinct, more indolent subset. The pattern of predominantly local, early failure without upfront RT is, as PNOC031 investigators have noted [33], unique to ETMR and strongly implicates the radiation-sparing design as suboptimal for the majority.

The prospective P-HIT trial and subsequent HIT2000-interim-registry (Juhnke et al., 2022) provide the most granular comparison of chemotherapy backbones in molecularly confirmed ETMR [15]. Thirty-five patients were identified (median age at diagnosis 2.9 years); nine (26%) had metastatic disease at presentation (M1 in 3 and M2/M3 in 6). Treatment was age-stratified and comprised carboplatin–etoposide induction, tandem high-dose chemotherapy (CARBO-ETO + HDCT), and response-stratified radiotherapy given for persistent disease, relapse, or progression rather than upfront; radiotherapy field (focal versus craniospinal) and dose varied with age and stage and were not uniformly reported. In the 30-patient analysis cohort (excluding four brainstem tumors and one untreated patient), five-year OS was 47% for CARBO-ETO + HDCT versus 8% for other treatments. Notably, on time-dependent Cox regression, the application of radiotherapy was not selected as an independent prognostic factor for OS in this cohort—a finding the authors attribute in part to the small numbers, the selective and delayed manner in which RT was delivered, and the competing risk of early progression before RT could be given. While CARBO-ETO + HDCT represents a meaningful advance over alternative chemotherapy-alone strategies, the absence of systematic early RT may contribute to a five-year OS ceiling that remains substantially below that achievable in other childhood CNS embryonal tumors of comparable stage.

### Early radiotherapy and the long-term survivor signal

The most compelling evidence for a survival benefit associated with early RT derives from two independent literature surveys with overlapping but nonidentical case repositories. Mayr and colleagues (2020) [17], in an analysis of nine institutional ETMR patients and a systematic survey of 237 published patients, reported that the only two institutional patients who received focal RT within approximately six weeks of diagnosis in both cases following gross total resection (GTR) remained alive and in complete remission at 50 and 56 months. All other institutional patients, for whom RT was deferred until the end of chemotherapy, recurred. Across the 237-patient cohort, only 26 individuals (11%) survived beyond 36 months without disease; 24 of 26 had received RT, and 10 of 26 had received early RT following GTR a proportion higher than the background rate in the full cohort. The authors emphasized that benefit appeared concentrated in patients undergoing both GTR and early focal RT, suggesting neither element alone was sufficient.

Jaramillo and colleagues (2018) [18], in an institutional series of seven ETMR patients treated with proton RT and a literature review of 204 cases, identified 18 long-term survivors (≥ 36 months); 17 of 18 (94%) had received RT as part of initial treatment and 14 of 18 had received CSI. Notably, all five institutional patients who progressed before the start of radiation failed at the primary tumor bed, a pattern consistent with local failure preceding systemic consolidation. This has direct implications for sequencing: if local failure occurs during extended chemotherapy and HDCT/ASCT, subsequent RT, however intensified, appears unable to rescue the outcome.

An important caveat is that “early RT” is not synonymous with a single radiation field: in Mayr et al., the survival signal was attributed specifically to early focal RT following GTR, whereas among the Jaramillo long-term survivors, 14 of 18 had received craniospinal irradiation, so the surveys conflate field, dose, and fractionation, which were heterogeneous and frequently unreported at the individual patient level. These surveys are further limited by heterogeneity of chemotherapy regimens, variation in RT technique, dose, and volume, and the selection biases inherent in retrospective data, including the survivorship bias that patients must survive progression-free long enough to reach late or consolidation-phase RT, so that comparisons of “early” versus “deferred” RT are confounded by baseline disease aggressiveness. Even so, their shared findings in that RT, and early RT especially, is a near-universal feature of long-term ETMR survival is among the more consistent observations in this literature. The multi-institutional SFCE series of 38 patients is consistent with this, finding that complete surgical resection, radiotherapy, and high-dose chemotherapy were each associated with improved outcome [7].

### The Rare Brain Tumor Registry: the largest molecularly confirmed cohort

The single largest molecularly confirmed ETMR dataset is the international Rare Brain Tumor Registry analysis reported by Khan and colleagues (2021), comprising 159 patients with centrally confirmed diagnoses (median age 26 months; C19MC alteration in 144 [91%], with 15 [9%] ETMR not otherwise specified) [9]. Most tumors were nonmetastatic (94 [73%] of 128 with staging data) and arose from both cerebral sites (84 [55%] of 154) and sites characteristic of other brain tumors (70 [45%]). Although not designed to test RT timing and although the registry recorded radiation field and dose but did not report the focal-versus-craniospinal breakdown or dose distribution within each treatment stratum, its treatment-stratified outcomes are directly relevant. Among patients treated with curative intent, EFS was 57% at six months and 31% at two years; OS was 29% at two years. Critically, two-year EFS was 0% in patients treated with conventional chemotherapy without RT, 21% (OS 30%) in those treated with HDCT and GTR without RT, and 66% in those treated with HDCT plus RT following GTR (44% after subtotal resection). The investigators concluded that prompt molecular diagnosis and intensive multimodal therapy including radiation could improve survival. By incorporating the largest molecularly confirmed cohort to date, these data substantially strengthen the cross-study signal favoring RT-inclusive consolidation and demonstrate that the dominant failure mode without radiation is early and local.

### The PPCR registry: focal proton RT and the limits of a timing analysis

The Pediatric Proton/Photon Consortium Registry (PPCR) provides the most recent multi-institutional data on ETMR outcomes following proton RT [29]. The full report (Krisch et al., 2025) described 20 patients enrolled from nine institutions between 2013 and 2021, of whom 18 were evaluable after excluding two with prior RT. Median age at RT was 3.0 years (range 1.7–12.2), and median follow-up was 55.5 months. The majority (16/18; 89%) received focal RT to a median dose of 54 Gy, while two patients received craniospinal irradiation (CSI, 30.6–36 Gy); 11 (61%) received systemic therapy with stem cell support. Four-year OS and EFS were 59.6% and 54.2%, respectively, and four-year local control was 81%, encouraging figures for this age group and consistent with effective local control from focal proton RT delivered as part of multimodality therapy. In this cohort, outcomes did not differ significantly by receipt of systemic therapy with stem cell support (OS *p* = 0.361; EFS *p* = 0.57), by progression prior to RT (*p* = 0.127; *p* = 0.18), or by a surgery-to-RT interval of ≥ 200 days (*p* = 0.35; *p* = 0.254). These comparisons are underpowered, and an important structural limitation is that the PPCR enrolls only patients who go on to receive RT: patients who never received RT, or who progressed and died during induction chemotherapy before RT could be delivered, are not captured, so the registry cannot by itself quantify the survival cost of RT deferral. The PPCR nonetheless demonstrates that focal proton RT achieves durable local control in ETMR and contributes a prospective, multi-institutional data point to the broader literature.

### Tandem versus single ASCT and the RT interface

The choice of single versus tandem ASCT adds further complexity. Tandem ASCT, as embodied by the HIT-MED protocol, involves sequential high-dose cycles each followed by stem cell rescue before any transition to radiation consolidation, substantially lengthening the interval between surgery and RT, typically to six months or beyond. Gevorgian and colleagues (2022) [20] reported on 50 patients with embryonal brain tumors (median age 39 months; medulloblastoma 28 [56%], ETMR 9 [18%], other histologies 13 [26%]), of whom 42 (84%) received tandem autologous HSCT by the HIT-MED protocol and 8 (16%) a single transplant; adjuvant radiotherapy was given in 25 (50%) and radiotherapy at relapse in a further 6 (12%). The tandem conditioning regimen comprised carboplatin–etoposide for the first transplant and thiotepa–cyclophosphamide for the second, with intraventricular methotrexate—broadly the same backbone used for tandem HDCT in the P-HIT trial (carboplatin/etoposide followed by cyclophosphamide/thiotepa). Five-year OS in the ETMR subgroup was 37%, versus 71% in the medulloblastoma subgroup a difference that, while not statistically significant given sample size (*p* = 0.07), is clinically substantial. The radiation-sparing five-year OS for the medulloblastoma subgroup was only 24%, confirming the contribution of RT even in that more radiosensitive histology, in which HDCT with craniospinal RT is well established [19]. The ETMR-specific outcomes in this tandem-ASCT series remain inferior to the early-RT-intensive approach suggested by the long-term survivor analyses. Durable remission without radiation has occasionally been documented including an infant with medulloepithelioma cured by high-dose chemotherapy and ASCT alone [21] and tandem high-dose regimens have improved outcomes for infants with embryonal CNS tumors more broadly [23]; ASCT can increasingly be delivered safely, including in outpatient settings [24]. Such radiation-free cures nonetheless remain the exception in ETMR.

### Pseudoprogression: a distinct complication of combined RT and HDCT/ASCT

The combination of RT and HDCT/ASCT in ETMR is associated with a specific, underrecognized complication: treatment-related pseudoprogression. Gutierrez-Quintana and colleagues [22] reported this phenomenon in all four consecutive ETMR patients treated with the CCG 99703 protocol incorporating HDCT/ASCT, in whom RT was administered either before (*n* = 3) or after (*n* = 1) HDCT. All four developed pseudoprogression new or enlarging lesions on MRI after treatment, accompanied by irritability, hypotonia, headache, and vomiting. Notably, the sequence of RT relative to HDCT did not prevent pseudoprogression given that it occurred regardless of whether RT preceded or followed the ASCT component.

Pseudoprogression is a diagnosis of exclusion; misinterpretation as true recurrence may prompt inappropriate salvage therapy. Its occurrence in every patient of this small, symptomatic series should be interpreted cautiously given the sample size, but it is a reminder to apply explicit pseudoprogression criteria when reviewing post-treatment imaging in ETMR patients who have received both modalities. Notably, survival in this series was among the best reported (three of four alive at a median 50.5 months), yet all survivors had severe neurocognitive impairment—underscoring that long-term survival in ETMR is not the same as uncomplicated cure.

### Late effects of radiotherapy and the proton therapy dimension

The neurocognitive consequences of early cranial RT in children under three years of age represent the primary argument against early RT in ETMR. CSI delivered at this age is associated with globally reduced intellectual function, working memory deficits, processing speed impairment, and educational difficulties persisting into adulthood [13]. These risks are dose-, volume-, and age-dependent and substantially greater than those seen with focal irradiation. Radiation late effects are not confined to cognition: sensorineural hearing loss and endocrine dysfunction (notably growth-hormone deficiency) are also well documented after cranial and craniospinal irradiation in young children. Ependymoma, like localized ETMR, typically treated with focal fields, is the most directly relevant comparator, and the neurocognitive burden of focal irradiation in this population is substantial enough that international trials now embed prospective cognitive testing into their design [35]; large proton ependymoma series report disease control comparable to photons, with the expected reductions in late toxicity still being quantified [36].

Proton beam radiotherapy offers a potentially meaningful mitigation through superior dose conformality and reduced integral dose to normal brain. Observational and prospective data from pediatric brain tumor cohorts suggest better preservation of cognitive function with protons than with conventional photon RT, particularly for focal volumes [25–27]. In ETMR, where tumors are typically supratentorial and localized, focal proton RT after GTR is theoretically attractive consolidating local control while minimizing exposure of the developing brain to scatter radiation. Mayr and colleagues noted that their two surviving patients, irradiated at ages 27 and 38 months, achieved neuropsychological outcomes within the average range at 3.6 years of follow-up [17]. While limited to two cases, this is consistent with the hypothesis that early focal proton RT, applied post-GTR in appropriately selected children beyond infancy, may achieve local control without unacceptable neurocognitive cost. PNOC031 operationalizes this with an age threshold for early focal RT of older than 12 months for infratentorial and older than 15 months for supratentorial tumors.

### The PNOC031 trial: addressing the central question

PNOC031 (NCT06861244) is the first international prospective trial designed to address the RT-timing question directly [33, 34]. Newly diagnosed patients, of any age, begin induction chemotherapy within 28 days of definitive surgery and are assigned to one of four cohorts by resection extent, metastatic status, and radiotherapy eligibility: cohort 1 (gross total resection, nonmetastatic, and RT-eligible) receives induction chemotherapy followed by early focal RT; cohort 2 (gross total resection, nonmetastatic, and RT-ineligible) receives high-dose chemotherapy with autologous stem-cell rescue; and cohorts 3 A and 3B (incompletely resected and/or metastatic disease) receive early focal RT or high-dose chemotherapy, respectively, according to RT eligibility. Radiotherapy eligibility is defined by age at planned radiation, older than 12 months for infratentorial tumors or older than 15 months for supratentorial tumors, rather than a single uniform threshold. The trial targets approximately 70 participants (PI: S. Mueller). Importantly, assignment is by clinical stratification rather than randomization, so between-cohort comparisons will remain susceptible to confounding by the very factors (age, resection extent, tumor location, and metastatic status) that determine cohort membership. The radiotherapy field is focal in the RT-containing arms; the specific prescribed dose and fractionation and the high-dose chemotherapy conditioning regimen for cohorts 2 and 3B are defined in the protocol and are not restated here.

The primary endpoint for cohort 1 is six-month PFS, chosen to assess whether early RT reduces the high rate of on-treatment relapse identified as a signature failure of radiation-sparing approaches. Secondary endpoints include two-year PFS and OS. PNOC031 incorporates extensive correlative studies (next-generation sequencing, CSF analyses, microbiome profiling, social determinants of health, and prospective neurocognitive assessment), providing the infrastructure to address neurodevelopmental outcomes alongside survival in a prospective framework. That the trial was designed around early RT, rather than further intensification of ASCT-centered consolidation, reflects growing doubt about radiation-deferred protocols in this disease.

## Discussion

A consistent signal emerges across the studies reviewed here: early radiotherapy following gross-total resection, given before or without protracted HDCT/ASCT, is associated with—though on current evidence cannot be shown to cause—improved long-term survival in ETMR. The signal is clinically important but rests on weak ground. It derives largely from retrospective and observational data, confounded by selection bias, protocol heterogeneity, and the extreme rarity of the disease.

Several lines of evidence support the early-RT hypothesis. The long-term survivor analyses of Mayr [17] and Jaramillo [18] independently show near-universal RT receipt among patients surviving beyond 36 months, with early timing overrepresented. The Rare Brain Tumor Registry [9], the largest molecularly confirmed cohort, shows two-year EFS of 0% without radiation in conventionally treated patients and 66% with HDCT plus RT after GTR, the most robust treatment-stratified comparison available. COG ACNS0334 data [16] show that local failure, the dominant recurrence pattern in ETMR, occurs frequently when radiation is withheld, and that salvage RT after local relapse is ineffective. Fourth, the PPCR registry [29] shows that focal proton RT achieves durable local control (four-year local control 81%) with encouraging survival, though in that small cohort outcomes did not differ significantly by RT timing or by receipt of HDCT, and the registry captures only patients who ultimately received RT. Fifth, the on-treatment relapse pattern characteristic of radiation-sparing protocols, described as unique to ETMR by the PNOC031 investigators [33], suggests that chemotherapy intensification alone cannot substitute for timely local consolidation.

The counterevidence is real but limited. The four ACNS0334 patients who achieved long-term remission without radiation [16] demonstrate that HDCT/ASCT alone may be curative in a biological subset [21] possibly those with DICER1-mutated rather than C19MC-amplified disease, or with other favorable molecular features [6] and the Rare Brain Tumor Registry similarly identified a minority surviving with HDCT and GTR without radiation [9]. The P-HIT/HIT2000 carboplatin–etoposide plus tandem-HDCT approach produced a five-year OS of 47% in the analysis cohort [15], meaningful in a disease where the historical benchmark is below 30%, albeit without systematic early RT and without radiotherapy emerging as an independent prognostic factor on time-dependent analysis. These data establish that HDCT/ASCT is not futile in ETMR and may represent the only viable consolidation strategy for patients ineligible for early RT. These apparently discordant observations are reconcilable if HDCT/ASCT is understood as a genuinely active systemic therapy whose value is independent of, rather than opposed to, timely local control. On this view the survival gain seen with CARBO-ETO + HDCT reflects more effective systemic disease control, while the inferior outcomes of radiation-sparing protocols reflect the loss of local control that follows when HDCT/ASCT is used specifically to defer radiation past the local control window. The harm attributed to “RT deferral,” in other words, is not a property of HDCT/ASCT itself but of the sequencing decision to substitute prolonged consolidation for early radiation; HDCT/ASCT and early focal RT are best regarded as complementary rather than competing, and the residual question PNOC031 must answer is which patients can safely receive one without the other.

The pseudoprogression seen in the CCG 99703 series [22] also merits comment. Its occurrence across different RT/HDCT sequences suggests it is not simply sequence-dependent but a consequence of the combined effect of these modalities on the tumor microenvironment and surrounding white matter; treatment-induced tumor maturation has likewise been described in occasional long-term survivors [30]. Clinicians and radiologists reviewing posttreatment imaging in ETMR patients who have received both modalities should apply explicit pseudoprogression criteria. Future prospective trials, including PNOC031, should prespecify MRI assessment protocols that distinguish pseudoprogression from true progression and collect longitudinal imaging characterizing its natural history.

The question of neurocognitive toxicity cannot be resolved by survival data alone. Long-term survival with profound neurocognitive impairment is not the same outcome as cure. Proton RT may shift this balance [25–27], though ETMR-specific data remain limited to small series. The prospective neurocognitive assessment built into PNOC031 should provide the most rigorous data to date on the functional cost of early RT in this age group and will be important for informing future guidelines.

**Fig. 2:**
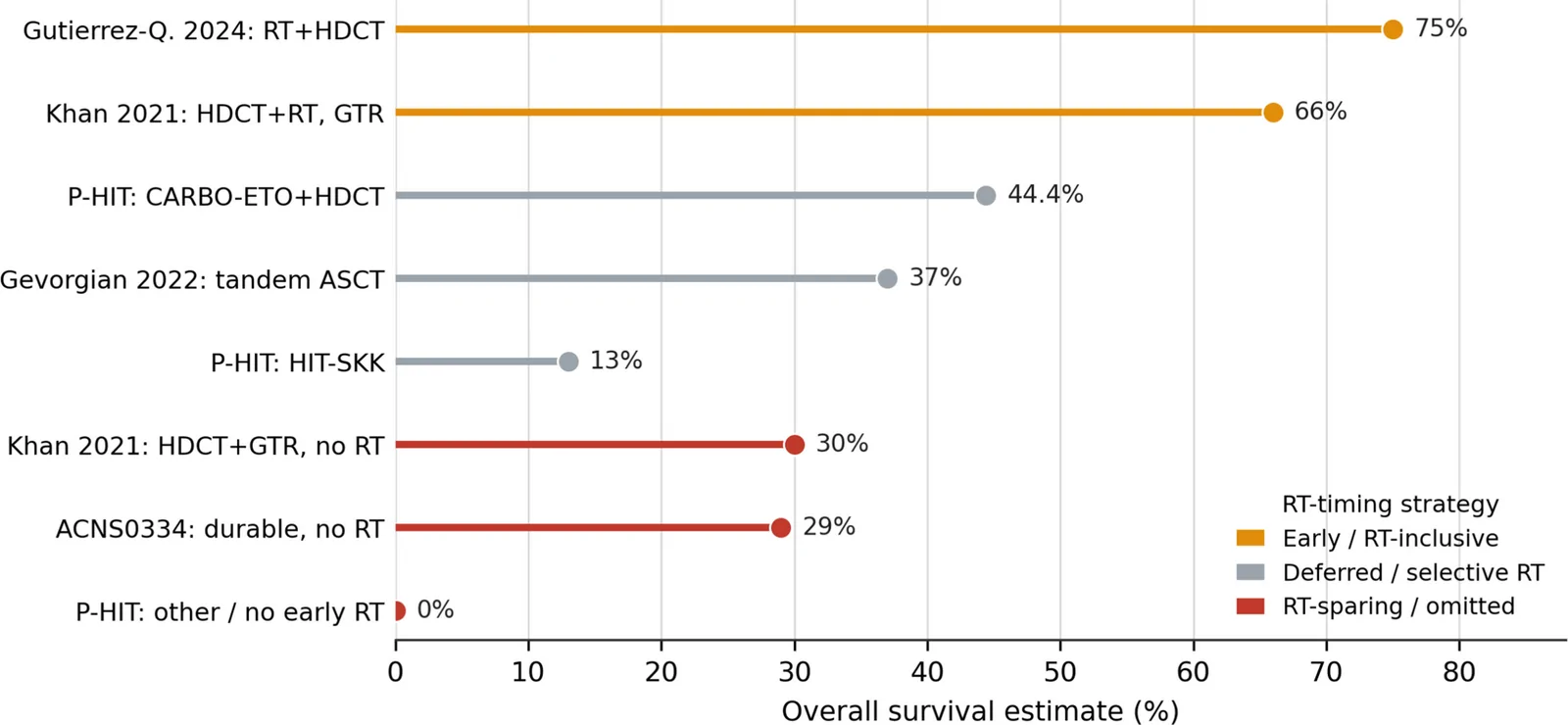
Study-level overall survival by radiotherapy-timing strategy. Each marker is a study-level overall- survival estimate extracted from the studies in this review, grouped by primary RT timing approach (early/RT-inclusive, deferred/selective RT, and RT-sparing/omitted). Because the source studies report survival at different landmarks, markers are separated into two panels— two-year and five-year overall survival—and the reported time point is labeled beside each marker; 95% confidence intervals are shown where the source reported them. Estimates from studies whose median follow-up does not reach the plotted landmark, or is unreported, are annotated as such, since Kaplan–Meier estimates beyond the median follow-up are unstable. Even within a panel, values are not directly comparable across studies owing to differences in cohort composition, era, and molecular confirmation, and should be read as a qualitative summary rather than a meta-analytic comparison

From a methodological standpoint, the limitations of this review are inherent to the field. Although the molecularly confirmed Rare Brain Tumor Registry now reports 159 patients [9] markedly enlarging the evidence base the total number of molecularly confirmed ETMR patients with extractable RT-timing data remains modest and is distributed across overlapping literature surveys, so apparent cohort totals cannot simply be summed. No randomized comparison of RT timing has been completed or, prior to PNOC031, attempted. Outcome data from different eras are conflated: a patient treated in 2005 with histological ETANTR may differ molecularly, and prognostically, from one diagnosed in 2020 with confirmed C19MC amplification. The 2021 WHO classification also introduced DICER1-mutated ETMR as a distinct subtype with potentially different radiosensitivity and prognosis [1, 6]. Notably, none of the cohorts reviewed here reported outcomes separately for DICER1-mutated ETMR, a subgroup whose baseline prognosis and radiosensitivity may differ from C19MC-amplified disease and whose omission could itself bias any apparent RT-timing signal; the proportion of historical, molecularly unclassified cases is also substantial across the nine cohorts, which is precisely the gap the prospective, molecularly driven PNOC031 design is intended to address. A further constraint is that the definition of “early” RT is not harmonized across sources, ranging from a strict biological window of within approximately six weeks of surgery (Mayr et al.) to broader registry thresholds such as ≤ 200 days from surgery (PPCR), so pooling across these incompatible parameters is not appropriate and no single quantitative threshold can be defended from these data. Because the long-term-survivor analyses also require patients to survive long enough to become eligible for late RT or for HDCT/ASCT, they are vulnerable to immortal time and survivorship bias; a landmark analysis of individual-patient data, were such data to be assembled, would be a more rigorous way to test the RT-timing hypothesis than the study-level synthesis possible here (Fig. 2). Future reviews and primary studies must stratify by molecular subtype, harmonize RT-timing definitions, and where possible use patient-level landmark methods to avoid masking biological heterogeneity within the ETMR umbrella [32].

## Conclusions

The timing of radiotherapy relative to HDCT/ASCT in ETMR is an important and understudied question on which the literature offers convergent but not definitive guidance. On balance, the available observational evidence is most consistent with a benefit from early focal RT following gross total resection in RT-eligible patients, with HDCT/ASCT retained as the primary consolidation strategy for those who cannot safely receive early radiation; this inference is necessarily provisional given the potential for selection bias. Using HDCT/ASCT to defer RT beyond the local control window appears to be associated with the high rates of early, local on-treatment failure that are characteristic of ETMR.

Pseudoprogression, an important complication of combined RT and HDCT/ASCT in this disease, should be anticipated and characterized prospectively. Neurocognitive outcomes, particularly when early RT is delivered at very young ages, need careful longitudinal assessment; proton RT is the most promising approach to reducing neurodevelopmental cost without sacrificing local control. PNOC031 will provide the first prospective comparative data on RT timing and is likely to shape the next generation of ETMR trials. Until those data are available, the evidence reviewed here supports early focal RT in eligible patients and argues against assuming that HDCT/ASCT intensity alone can compensate for delayed or omitted radiation in this aggressive tumor.

## Data Availability

No datasets were generated or analysed during the current study.
